# Metallic Sn‐Based Anode Materials: Application in High‐Performance Lithium‐Ion and Sodium‐Ion Batteries

**DOI:** 10.1002/advs.201700298

**Published:** 2017-09-22

**Authors:** Hangjun Ying, Wei‐Qiang Han

**Affiliations:** ^1^ School of Materials Science and Engineering Zhejiang University Hangzhou 310027 P. R. China; ^2^ Ningbo Institute of Materials Technology & Engineering Chinese Academy of Sciences Ningbo 315201 P. R. China; ^3^ College of Materials Science and Opto‐Electronic Technology University of Chinese Academy of Sciences 19 A Yuquan Rd Shijingshan District Beijing 100049 P. R. China

**Keywords:** alloying, lithium‐/sodium‐ion batteries, metallic Sn‐based anode, size control, structure design

## Abstract

With the fast‐growing demand for green and safe energy sources, rechargeable ion batteries have gradually occupied the major current market of energy storage devices due to their advantages of high capacities, long cycling life, superior rate ability, and so on. Metallic Sn‐based anodes are perceived as one of the most promising alternatives to the conventional graphite anode and have attracted great attention due to the high theoretical capacities of Sn in both lithium‐ion batteries (LIBs) (994 mA h g^−1^) and sodium‐ion batteries (847 mA h g^−1^). Though Sony has used Sn–Co–C nanocomposites as its commercial LIB anodes, to develop even better batteries using metallic Sn‐based anodes there are still two main obstacles that must be overcome: poor cycling stability and low coulombic efficiency. In this review, the latest and most outstanding developments in metallic Sn‐based anodes for LIBs and SIBs are summarized. And it covers the modification strategies including size control, alloying, and structure design to effectually improve the electrochemical properties. The superiorities and limitations are analyzed and discussed, aiming to provide an in‐depth understanding of the theoretical works and practical developments of metallic Sn‐based anode materials.

## 1 Introduction

The environmental pollution and energy crisis have raised growing concerns across the world in recent decades due to the rapid industrialization and increasing demands for energy. To solve these issues, it is urgent that green renewable energy sources are exploited and popularized. Consequently, high‐performance energy storage devices have become a hot research topic in recent years. Rechargeable ion batteries are supposed to have the capability to carry that responsibility, given their appealing advantages of high energy density, rational working voltage and good cyclability.^1^, ^2^, ^3^

Lithium‐ion batteries (LIBs) have gradually become the dominant energy storage devices since Sony first commercialized them in 1991, and they are now showing great potential to alter the vehicle situation. However, the current energy density of LIBs (150–200 Wh kg^−1^) is still insufficient to promote their universal application in electric vehicles (EVs), wearable electronic devices, smart grid systems, etc.^4^, ^5^ Carbon‐based materials are the current mainstream anodes on the market, however they have been faced with a bottleneck due to their low theoretical capacity (372 mA h g^−1^ for graphite). As the capacities of cathode materials have been constantly progressing, it is vital that we develop new anode materials with higher capacities. Although having similar electrochemical energy storage mechanism, sodium‐ion batteries (SIBs) usually have poorer electrochemical properties than that of LIBs because of the much bigger size of Na^+^ (0.59 Å for Li^+^ and 1.02 Å for Na^+^ in radius). This big Na^+^ size results in poor kinetic performance and large volume fluctuations.^6^, ^7^ Hence SIBs have inspired much less research enthusiasm than LIBs in the past. However, as lithium resources are heavily consumed, SIBs have drawn increasing attention because of the abundant global sodium reserves and their relatively low cost.^3^, ^5^, ^8^ It is reckoned that about 7.9 million tons of lithium metal will be consumed when 50% of gasoline‐powered vehicles in the world are replaced by electric vehicles.^3^ The rareness of lithium sources highlights the competitive advantages of SIBs for large scale application.

**Figure 1:**
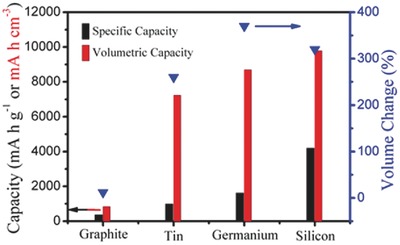
Capacity and volume change comparison of IVA group elements.

Besides carbon, other IVA group elements (especially Si, Ge, and Sn) have attracted great research interest owing to their ultrahigh theoretical capacities.^9^, ^10^, ^11^ **Figure** **1** shows the capacity and volume change comparison of IVA group elements. Although the theoretical specific capacity of Sn is not the highest among them, the volumetric specific capacity of Sn is quite close to those of Si and Ge. Moreover, the Sn and Sn‐based compounds have been heavily researched due to their merits of high availability, low cost, and high electrical conductivity.^12^, ^13^, ^14^, ^15^, ^16^ In 2005, Sony corporation announced the commercialization of a new type of lithium‐ion battery, named “Nexelion,” which was the first to use an amorphous Sn–Co–C composite as a negative electrode, and led to a 30% volumetric capacity increase over conventional LIBs.^17^, ^18^ This breakthrough had ignited great passion from researchers in studying metallic Sn‐based anodes.

Based on the alloying reactions with Li and Na ions, Sn can deliver quite high specific capacities as anodes in lithium‐ion and sodium‐ion batteries. The theoretical specific capacity of Sn reaches 994 mA h g^−1^ for LIBs according to Li_22_Sn_5_, and 847 mA h g^−1^ for SIBs according to Na_15_Sn_4_.^19^, ^20^ However, the drastic volume changes during Li and Na ions insertion/extraction (260% for LIBs and 420% for SIBs) always bring irreconcilable inner stress, and result in a series of negative consequences: active material particles pulverize and lose electrical connection with the current collectors, the newly formed surfaces constantly consume the Li and Na sources by forming solid electrolyte interphase (SEI) film, aggregation of particles results in poor kinetics of the electrodes, etc.^12^ The volume expansion problem cannot be eradicated completely, hence the main principles of Sn‐based anode modification include the alleviation of volume expansion by reducing the particle sizes and introducing either accommodation space or buffer agents, by either direct or indirect methods; such as the preparation of nanoporous materials, dispersing Sn in a carbon matrix and fabricating Sn‐based alloys.^21^, ^22^, ^23^, ^24^

The metallic Sn‐based materials have common features in both synthesis and physicochemical/electrochemical properties. This review will summarize the state‐of‐the‐art preparation methods, characteristics, and electrochemical performances of metallic Sn‐based anode materials from recent years. We focus on the research of metallic Sn‐based anodes in LIBs and SIBs from three aspects: size control of Sn, alloying modification, and structure design of Sn‐based composite materials. We expect that this comprehensive and up‐to‐date summary can provide a reference for the development and application of Sn‐based anode materials.

## 2 Size Control of Sn Anodes

### 2.1 Nanocrystallization of Bare Sn Anodes

Nanocrystallization is an important modification method to improve the cycling stability of Sn‐based materials. Remarkably, nanoparticles can decrease the absolute volume change of every single particle with the rate of third power of particle diameter. As a result, the absolute strain is efficiently mitigated and the structural stability of the material is greatly enhanced.^25^, ^26^, ^27^ In addition, nanoparticles shorten the charge‐diffusion route for both ions and electrons and supply abundant electrochemically active sites.^28^, ^29^, ^30^ The characteristic diffusion time of ions in active electrode materials can be represented as: τ = *L* ^2^/*D*, where *L* is the ion diffusion distance, *D* is the ion diffusion coefficient. The diffusion time (τ) decreases with the square of diffusion distance (*L* ^2^), so the rate capability can be effectively improved by reducing the particle size.^31^ Furthermore, the interspace among nanoparticles can accelerate the infiltration of electrolyte and provide reserved buffer space for volume expansion. However, it still remains significant challenges to control the particle size of Sn using simple methods because of the low melting point and coalescence features of Sn.

**Figure 2:**
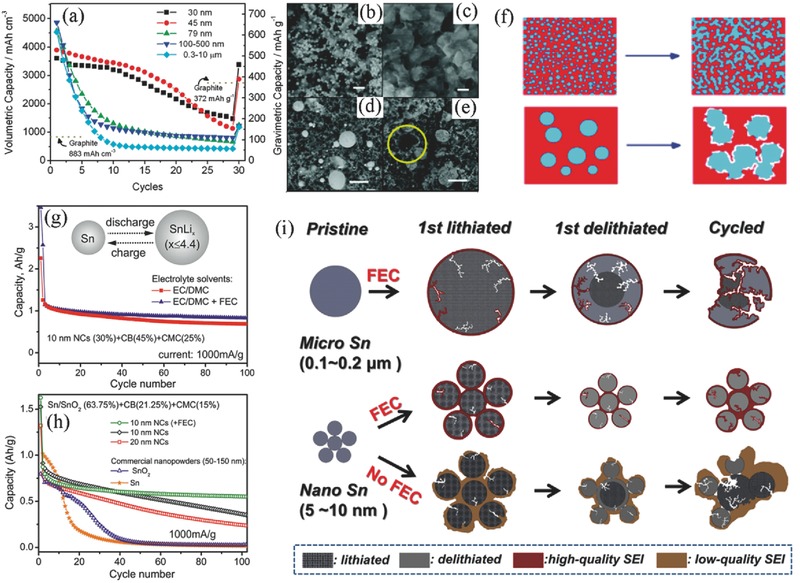
a) Cycling performance of Sn/SnO*_x_* particles with different size distribution. SEM images of electrodes for b,c) 45 nm nanospheres before and after cycling, respectively; d,e) 0.3–10 µm particles before and after cycling, respectively. The scale bars are 200 nm for a,b), and 2 µm for c,d). f) Schematic illustration of the electrodes surface topography evolution upon cycling. Reproduced with permission.^32^ Copyright 2010, American Chemical Society. g) Cycling performance of anodes containing 30 wt% Sn/SnO_2_ NCs in electrolyte with/without FEC (calculating based on the content of active material). h) Cycling performance comparison of Sn/SnO_2_ NCs and commercial samples. Reproduced with permission.^33^ Copyright 2013, American Chemical Society. i) Schematic illustration of the formation and degradation mechanisms of high‐/low‐quality SEI films in nano‐/micro‐Sn electrodes with/without FEC additive. Reproduced with permission.^34^ Copyright 2015, Elsevier.

In order to investigate the effect of Sn particle size to the cycling performance, Wang et al.^32^ synthesized monodisperse Sn particles ranging from 30 to 1200 nm through a modified polyol wet‐chemistry process. As shown in **Figure** **2**a, Sn nanospheres (30 and 45 nm) showed better electrochemical performance than that of microsized samples. The microscopic morphology characterization revealed that the microstructural evolution of Sn electrodes depended on the particle sizes. The nanoscale Sn particles suffered from aggregation after cycling (Figure 2b,c). Instead, some microsized particles coarsened after cycling and split away off the matrix, which led to the rapid deterioration of capacity (Figure 2d,e). As schematic shows in Figure 2f, the decline of nanoscale particles was mainly due to the limitation of kinetics resulting from the particle aggregation. In contrast, the loss of effective active materials in microsized sample resulted in unrecoverable capacity fading.

Nanostructure Sn can promote the homogeneous lithiation/delithiation in a single particle and alleviate the volume mismatch, thus avoiding crack propagation and improving structural stability. However, because of the soft nature of Sn, aggregation inevitably occurs after cycling even in 30 nm Sn particles. Further control of the Sn particle size is pursued by researchers. Kravchyk et al.^33^ synthesized monodisperse Sn and Sn/SnO_2_ nanocrystals with main sizes tunable from 9 to 23 nm. The performance comparison result showed that the 10 nm Sn/SnO_2_ NCs displayed better cycling ability than that of the 20 nm sample and the commercial Sn and SnO_2_ nanopowders (Figure 2h). As shown in Figure 2g, the addition of fluoroethylene carbonate (FEC) could help to improve the cycling performance by forming high‐quality SEI films and restraining side reactions.^34^, ^35^, ^36^

The effects of FEC addition on the SEI formation and Sn particles protection were comprehensively investigated by Eom et al.^34^ As schematically illustrated in Figure 2i, FEC could impede the superfluous SEI formation and reduce the irreversible Li^+^ exhaustion during cycling. The nano‐Sn (5–10 nm) with FEC cycled steadily after the initial 15 cycles even at a high rate of 320 mA g^−1^, in contrast, nano‐Sn without FEC underwent a continuous capacity fade between 40 and 320 mA g^−1^. However, the mitigating effect on capacity fade was not observed in microsized Sn (0.1–0.2 µm), which rapidly failed after 15 cycles even with FEC. The huge volume expansion caused cracks and the long ion diffusion path aggravated the crack propagation, leading to the particles pulverization and continuous SEI formation on the newly exposed surfaces. As a result, the protection mechanism of high quality SEI is invalid in microparticles.

**Figure 3:**
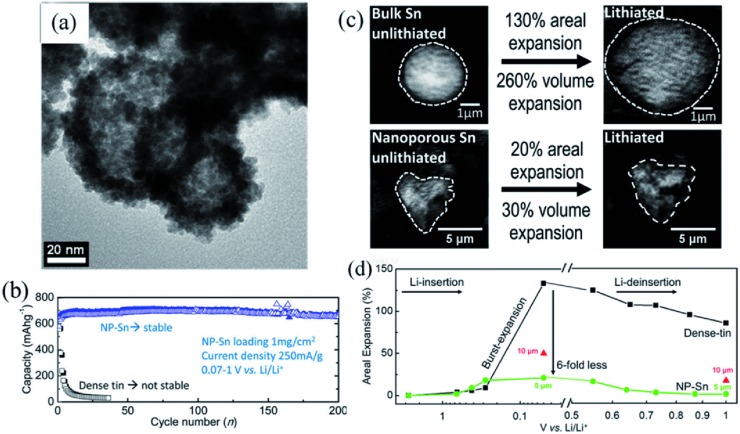
a) TEM image of NP‐Sn. b) Cycling performance comparison of NP‐Sn and dense Sn at current density of 250 mA g^−1^. c) Transmission X‐ray microscope absorption images of NP‐Sn and dense Sn at unlithiated and lithiated states. d) Percent areal expansion of dense Sn, and NP‐Sn at different lithiation voltages. Reproduced with permission.^21^, ^27^ Copyright 2017, American Chemical Society.

Another advantage of nanoscale Sn is that the abundant clearance space can effectively accommodate the drastic volume changes.^37^, ^38^ Cook et al.^21^, ^27^ prepared nanoporous Sn (NP‐Sn) powders by selective dealloying of Sn–Mg binary system according to Equation (1). The NP‐Sn had unique ligament morphology, which was composed of clustered ≈5 nm Sn nanocrystals (**Figure** **3**a). Based on the synchrotron‐based transmission X‐ray microscopy, the authors found that the inner space of NP‐Sn could effectively accommodate the volume change. Impressively, NP‐Sn underwent a sixfold smaller lithiation areal expansion than dense Sn, with only ≈20% areal expansion or ≈30% volume expansion after lithiation (Figure 3c). Moreover, the NP‐Sn particles could contract back to its original size after delithiation (Figure 3d). In sharp comparison to the quick collapse of dense Sn after 5 cycles, the NP‐Sn cycled above 200 cycles without obvious decay (Figure 3b)

(1) $$\mathrm{Mg}\left(s\right)\;\;+\;\;2{\mathrm{NH}}_{4}^{+}\left(\mathrm{aq}\right)\;\;\to \;\;{\mathrm{Mg}}^{2+}\left(\mathrm{aq}\right)\;\;+\;\;H_{2}\left(g\right)\;\;+\;\;2{\mathrm{NH}}_{3}\left(\mathrm{aq}\right)$$

In addition, nanoscale Sn provides an ideal platform for studying the mechanisms of energy storage.^15^, ^24^, ^39^, ^40^, ^41^, ^42^, ^43^, ^44^ For example, Im et al.^39^ synthesized Sn, SnS, and SnO_2_ nanocrystals (NCs) by gas‐phase laser photolysis and investigated their phase evolution during lithiation/delithiation processes. All three samples could produce cubic phase α‐Sn NCs during cycling, the α‐Sn NCs preserved the crystal structure upon lithiation/delithiation processes and hence increased the electrical conductivity. The SnS NCs exhibited the best rate capability, which had the strongest transformation tendency toward α‐Sn. Wang et al.^45^ used in situ TEM to study the electrochemical sodiation mechanism of Sn nanoparticles in a nanobattery configuration. It was found that pristine Sn could be sodiated in two steps. In the first step, amorphous Na*_x_*Sn (*x* ≈ 0.5) is formed through migrating phase boundary alloying reaction, with a mild volume expansion of 60%. In the second step, amorphous Na_2_Sn is further sodiated to amorphous Na_9_Sn_4_ and Na_3_Sn, and finally to crystalline Na_15_Sn_4_, with a final volume change of 420%.

In summary, size control has been proven to be an effective way to mitigate the pulverization and prolong the cycling life of Sn‐based electrode materials. It was found that there exists a critical size below which the fracture of Si particles will be eliminated, because amorphization of Si during lithiation dissipates a part of the energy involved in the volume change.^46^ However, the intermediates produced during Sn lithiation are crystalline, and the strain inevitably accumulates between different crystalline phases.^47^ Xu et al.^24^ found that significant mechanical damage still occurred even in 10 nm Sn crystals, revealing that size control alone was not sufficient to eradicate the pulverization problem. Moreover, some negative factors of nanocrystallization are considerable issues, such as the high‐cost and complex preparation methods of nano‐Sn,^24^, ^33^ large interface contact resistance among nanocrystals,^27^ low coulombic efficiency caused by the high surface areas and side reactions,^32^ low compaction density of nanomaterials and the inflammable and explosive characteristics of nanometallic materials. Thereby, size reduction of bare Sn may not be a feasible approach to the practical utilization of Sn anodes.

### 2.2 Sn Nanoparticles in Carbon Matrix

In order to take full advantage of being small in size and avoid the negative effects of nanocrystallization, researchers try to control the Sn size by dispersing it in a carbon matrix.^6^, ^22^, ^26^, ^48^, ^49^, ^50^, ^51^, ^52^, ^53^, ^54^, ^55^ The stable and flexible carbon matrix can effectively suppress the tendency of grain aggregation and growth during preparation, thus controlling the Sn particle sizes with a relatively simple process at low cost. In addition, the carbon matrix prevents the direct contact of Sn and electrolyte and greatly avoids the adverse side reactions, thereby improving the coulombic efficiency.^52^ The carbon matrix also works as a high‐efficiency conducting medium and enhances the rate performance of electrode materials.

People have varied the carbon precursors to produce the carbon matrix, including micromolecular organics,^22^, ^26^, ^49^, ^50^, ^52^, ^56^ polymer,^6^, ^53^, ^57^, ^58^, ^59^ saccharides,^48^, ^51^, ^60^, ^61^, ^62^ resins,^55^, ^63^, ^64^ graphene,^65^, ^66^, ^67^ etc. Derrien et al.^49^ reported a synthesis of nano‐Sn (with a small amount of SnO_2_) embedded in a carbon matrix. Resorcinol and formaldehyde were used as the starting materials to form hydrogel as carbon precursors. The Sn source Tributylphenyltin was impregnated by stirring with carbon precursors. The Sn particles size was about 50 nm at the surface of carbon matrix, but sub‐10 nm in the bulk. This Sn–C composites showed excellent cycling stability, maintaining 500 mA h g^−1^ for over 200 cycles at 0.8 C.

The nitrogen‐doped carbon has been found to be a very efficient matrix in restraining particle growth and coalescence of Sn. Ultrasmall Sn particles (even Sn quantum dots) are usually obtained when N‐doped carbon is used as the matrix. N‐doping can add defects in the carbon, which is favorable as it increases the distribution density of Sn.^57^, ^68^ The interfacial Sn—N—C and/or Sn—O—C bonds probably form between Sn and N‐doped carbon and pin the Sn particles to the carbon, as a result, the aggregation of Sn is thoroughly inhibited.^68^ Similarly, the M—O—C bonds formed between metallic oxides (MO) and surface functional groups on carbon have been reported previously.^69^, ^70^, ^71^ These interface interactions can effectually anchor the active materials to the carbon matrix, and inhibit particles growth and aggregation. Therefore, these covalent bonds are always beneficial to the electrochemical properties. Furthermore, N‐doping further enhances the electrical conductibility of the carbon matrix.^72^

**Figure 4:**
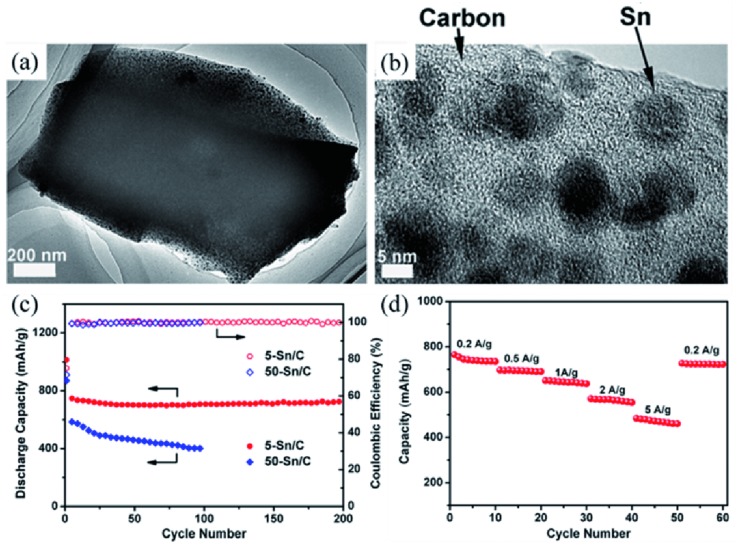
a,b) TEM and HRTEM images of 5‐Sn/C composite. c) Cycling performance comparison of 5‐Sn/C and 50‐Sn/C composites. d) Rate performance of 5‐Sn/C composite. Reproduced with permission.^52^ Copyright 2013, American Chemical Society.

For example, Chen and co‐workers^52^ prepared ultra‐small Sn nanoparticles embedded in nitrogen‐doped porous carbon by using a divalent Sn complex, Sn(Salen) as precursor. Sn was homogenously distributed in the complex at molecular dimension. As shown in **Figure** **4**a,b, the metal cation was in situ reduced to form uniform Sn nanoparticles about 5 nm (denoted as 5‐Sn/C). Benefiting from the tight embedment of Sn in carbon, the composite displayed a high initial coulombic efficiency of 75%. The 5‐Sn/C showed excellent structural stability, retaining 722 mA h g^−1^ after 200 cycles at 0.2 A g^−1^ (Figure 4c). Furthermore, a high reversible capacity of 480 mA h g^−1^ was still obtained at 5 A g^−1^ (Figure 4d). This remarkable electrochemical performance could be attributed to the elegant combination of ultrasmall Sn and the conductivity enhanced porous carbon network skeleton, which efficiently solved the problems of pulverization and particle aggregation of Sn particles.

**Figure 5:**
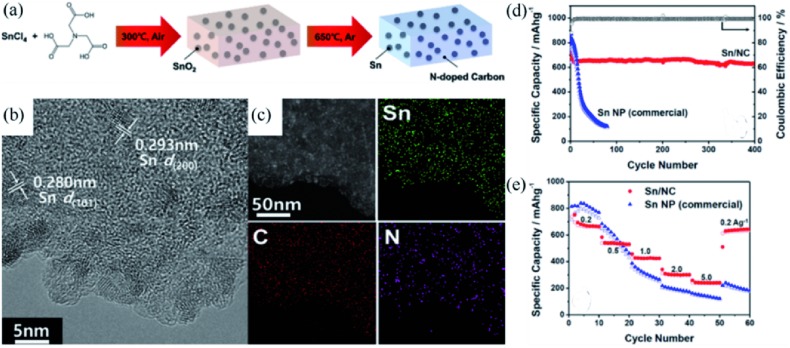
a) Schematic diagram of the synthesis process of Sn/NC. b,c) TEM image and elemental mapping of Sn/NC. d) Cycling performance of Sn/NC and commercial Sn NP at 200 mA g^−1^. e) Rate performance of Sn/NC and commercial Sn NP. Reproduced with permission.^22^ Copyright 2016, American Chemical Society.

Similarly, as schematically presented in **Figure** **5**a, Mullins and co‐workers^22^ prepared nanostructured Sn (≈3.5 nm)/nitrogen‐doped carbon composites (Sn/NCs) by using nitrilotriacetic acid as a carbon and nitrogen source. The nitrilotriacetic acid could chelate the metal ions and ensure the uniform and tight distribution of Sn in the matrix (Figure 5b,c). The N‐doped carbon, or its precursor, is a much better reducing agent than undoped carbon, allowing a lower reduction temperature during sintering, which not only saves energy, but also inhibits the growth of Sn. Owning to the uniform distribution of ultra‐small Sn in the high‐conductive N‐doped carbon, the Sn/NC anodes presented both improved capacity stability and rate performance, in sharp contrast to the commercial Sn particles (Figure 5d,e).

## 3 Alloying Modification of Sn‐Based Anode Materials

In accordance with the properties of Sn, introducing other metals to form Sn intermetallic compounds is perceived as another promising method to overcome the drawbacks of pulverization and coalescence of bare Sn anodes. The introduced metals can evenly and closely combine with Sn through metallic bonds, supplying a soft buffering framework and conductive network, thereby improving the electrochemical performances. As shown in **Table** **1**, we have summarized some state‐of‐the‐art progress in Sn‐based alloy materials for LIBs and SIBs.

**Table 1:** Summary of the structure, electrochemical performance, and preparation method of some typical Sn‐based alloy materials

Property of alloying metals	Sn‐based alloy materials	Structure	Electrochemical performance	Preparation method
Inactive metals	CoSn*_x_*@C–PAn^80^	CoSn*_x_* alloy sealed in carbon shell and embedded in N‐doped porous graphitic carbon matrix	LIBs: 2044 mA h g^−1^ after 100 cycles at 0.2 A g^−1^, 1256 mA h g^−1^ after 1000 cycles at 10A g^−1^	In situ polymerization and annealing process
	Sn–Ni–Cu alloy@carbon^123^	Core–shell Sn–Ni–Cu alloy@ carbon nanorods	LIBs: 100% capacity retention about 450 mA h g^−1^ over 400 cycles at 450 mA g^−1^, excellent rate performance up to 9000 mA g^−1^	Pulse nanoelectrodeposition
	Ni–Sn alloy^23^	3D highly porous Ni scaffold supported Ni–Sn alloy structure	LIBs: 895 mA h g^−1^ at 0.5 C and maintained ≈750 mA h g^−1^ after 200 cycles	Electrodeposition
	Co_3_Sn_2_@Co nanoparticles^124^	Core–shell Co_3_Sn_2_@Co nanoparticles pinned on N‐Doped graphene	LIBs: 1615 mA h g^−1^ at 250 mA g^−1^ after 100 cycles, 793.9 mA h g^−1^ at 2500 mA g^−1^	Hydrothermal and annealing process
	Sn_2_Fe/Sn_2_Co^77^	Uniform cubic shaped particles	LIBs: 510 mA h g^−1^ after 50 cycles at 50 mA g^−1^, 443 mA h g^−1^ at 1000 mA g^−1^	Reduction‐thermal diffusion alloying reaction
	Co–Sn intermetallic electrodes^83^	Highly ordered mesoporous Co*_x_*Sn*_y_* framework	LIBs: ≈530 mA h g^−1^ after 50 cycles at 0.1 C	Nanoreplication method
	Sn–Cu nanocomposite^125^	100 ± 34 nm nanoparticles composed of multiple small monocrystals	SIB: 420 mA h g^−1^ at 0.2 C and maintained 97% after 100 cycles, 126 mA h g^−1^ at 1694 mA g^−1^	Surfactant‐assisted wet chemistry method
	MnSn_2_ electrodes^93^	Ragged morphology with particle sizes from nanometers to micrometers	SIBs: 400 mA h g^−1^ for over 50 cycles at 18.35 mA g^−1^	Mechanosynthesis method
	FeSn_2_‐carbonaceous composites^76^	FeSn_2_ particles embedded in a carbon matrix	SIBs: 333 mA h g^−1^ after 100 cycles at 100 mA g^−1^	Hydrothermal route and ball‐milling
Active metals	Sn_78_Ge_22_@Carbon^126^	Sn_78_Ge_22_@carbon core–shell nanowires	LIBs: 1107 mA h g^−1^ at 0.3 C and maintained 94% after 45 cycles, 93% capacity retention at 8 C	Thermal annealing method
	SnSb nanocrystals^108^	≈20 nm SnSb nanocrystals	LIBs: >700 and >600 mA h g^−1^ after 100 cycles at 0.5 C and 4 C SIBs: >350 and >200 mA h g^−1^ at 1 C and 20 C	One‐pot wet chemical reduction
	SnSb/C composite nanofibers^127^	SnSb nanoparticles embedded in electrospun carbon nanofibers with porous structure	LIBs: 659 mA h g^−1^ after 150 cycles at 50 mA g^−1^, 354 mA h g^−1^ at 1600 mA g^−1^	Electrospinning and carbothermal method
	CNT‐encapsulated Sn–Sb nanorods^128^	Integrated coaxially core–shell structure with an Sn–Sb core and a carbon nanotube shell	LIBs: ≈700 mA h g^−1^ after 80 cycles at 0.2 C	Chemical vapor deposition
	Sn–Ge alloy^107^	≈8 µm flake‐like ribbons of Sn–Ge alloy	LIBs: 1000 mA h g^−1^ after 60 cycles at 0.1 C, 500 mA h g^−1^ at 5 C	Melt spinning
	Ge_1–_ *_x_*Sn*_x_* alloy nanocrystals^106^	Nanocrystals with an average size of 10 ± 1 nm	LIBs: 1010 mA h g^−1^ after 50 cycles at 0.1 C, 650 mA h g^−1^ at 5 C	Gas‐phase laser photolysis reaction
	Sn–Ge alloys^105^	Nanostructured films with column diameter domains of 500–800 nm	SIBs: ≈650 mA h g^−1^ after 100 cycles at 0.5 C	Vacuum deposition

### 3.1 Sn Alloyed with Electrochemically Inactive Metals

Electrochemically inactive metals cannot undergo lithiation/sodiation with Li^+^/Na^+^, for example, the electrochemical reaction of FeSn_2_ in LIBs can be described as follows^73^

(2) $${\mathrm{FeSn}}_{2}\;\;+\;\;8.8{\mathrm{Li}}^{+}\;\;+\;\;8.8e^{-}\;\;\to \;\;2{\mathrm{Li}}_{4.4}\mathrm{Sn}\;\;+\;\;\mathrm{Fe}$$

(3) $${\mathrm{Li}}_{4.4}\mathrm{Sn}\;\;↔\;\;\mathrm{Sn}\;\;+\;\;4.4{\mathrm{Li}}^{+}\;\;+\;\;4.4e^{-}$$

Thus the inactive metals are ideal buffer agents to the volume expansion of electrochemically active Sn. Meanwhile, the introduced metals can enhance the conductivity and improve the rate ability. For example, Thackeray and co‐workers found that excess Cu in Cu_6_Sn_5_ could improve the utilization of Sn. The excess Cu might lead to more finely divided Sn, and accelerate the diffusion of lithium. Furthermore, the excess Cu could serve as an additional conducting matrix and volume buffer agent.^74^, ^75^ In 2005, Sony corporation applied the amorphous Sn–Co–C composites as negative electrodes for its new‐type lithium‐ion batteries, named “Nexelion.”^17^, ^18^ This breakthrough had ignited great passion from people in researching of Sn‐based alloys. To date, a series of Sn‐based intermetallics alloyed with inactive metals have been investigated as potential anodes for LIBs and/or SIBs, including Fe–Sn,^73^, ^76^, ^77^, ^78^ Co–Sn,^79^, ^80^, ^81^, ^82^, ^83^, ^84^, ^85^ Cu–Sn,^86^, ^87^, ^88^, ^89^ Ni–Sn,^23^, ^90^, ^91^, ^92^ Mn–Sn,^93^, ^94^, ^95^ La–Sn,^96^, ^97^ Ce–Sn,^98^ Cr–Sn,^99^, ^100^ etc.

**Figure 6:**
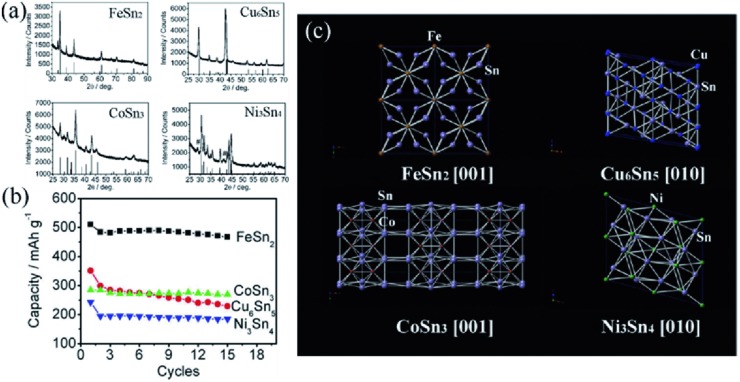
a) XRD patterns of FeSn_2_, Cu_6_Sn_5_, CoSn_3_, and Ni_3_Sn_4_ intermetallic nanospheres. b) Capacity and cycling performance of M–Sn (M = Fe, Cu, Co, and Ni) intermetallic compounds at 20/C (based on the theoretical capacities) in the voltage range of 0.05–1.5 V. c) Crystal structures of FeSn_2_, Cu_6_Sn_5_, CoSn_3_, and Ni_3_Sn_4_ from specific view directions. Reproduced with permission.^101^ Copyright 2010, American Chemical Society.

Wang et al.^101^ had systematically studied the influences of alloying metals to the anode performances, including M–Sn (M = Fe, Cu, Co, and Ni). These intermetallics were synthesized in similar methods and had similar morphological features. **Figure** **6**a displays the XRD patterns of these intermetallics. It was found that the reversible capacities of these morphologically controlled intermetallics were not directly dictated by their theoretical capacities. The theoretical capacities are CoSn_3_ (852 mA h g^−1^) > FeSn_2_ (804 mA h g^−1^) > Ni_3_Sn_4_ (725 mA h g^−1^) > Cu_6_Sn_5_ (605 mA h g^−1^), while the practical capacities followed FeSn_2_ > Cu_6_Sn_5_ ≈ CoSn_3_ > Ni_3_Sn_4_ (Figure 6b). A moderate cathodic peak at ≈0.8 V was observed in the first cyclic voltammogram (CV) scan of FeSn_2_, indicating a high‐quality solid electrolyte interface (SEI) film was formed in FeSn_2_. In contrast, Cu_6_Sn_5_ and Ni_3_Sn_4_ did not show the cathodic peaks related to the SEI formation and CoSn_3_ showed a strong and sharp peak, suggesting Cu_6_Sn_5_ and Ni_3_Sn_4_ might not protected by SEI film and that CoSn_3_ was coated tightly by a thick SEI film, both generating an adverse effect to their electrochemical performances. This might partially account for the better electrochemical performance of FeSn_2_. To comprehend from the crystal structure view (Figure 6c), FeSn_2_ crystal has open channels parallel to [001] direction, which are surrounded by adjacent Sn atoms, this favorable structure promotes the penetration of Li^+^ into the grains and facilitates the alloying reaction between Li^+^ and Sn. In comparison, the channels in the other intermetallic compounds are smaller and distorted (i.e., Cu_6_Sn_5_ and Ni_3_Sn_4_), or lack accessible Sn layers along the open channels (i.e., CoSn_3_). As a result, poorer capacities were delivered in these intermetallic systems.

**Figure 7:**
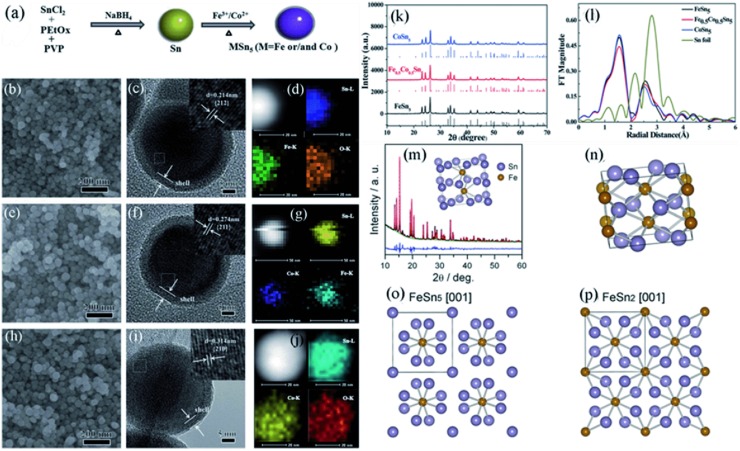
a) Synthesis process of MSn_5_ (M = Fe, Co, and Fe_0.5_Co_0.5_) intermetallic nanospheres. b,e,h) SEM; c,f,i) HRTEM; and d,g,j) elemental mapping images of FeSn_5_, Fe_0.5_Co_0.5_Sn_5_, and CoSn_5_ intermetallic nanospheres. k) Synchrotron XRD patterns of MSn_5_ (M = Fe, Co, and Fe_0.5_Co_0.5_). l) Synchrotron XAFS profiles of MSn_5_ (M = Fe, Co, and Fe_0.5_Co_0.5_) and Sn foil. Reproduced with permission.^102^ Copyright 2015, Royal Society of Chemistry. m) Synchrotron XRD pattern and the Rietveld refinement of Fe_0.74_Sn_5_, the inset shows the crystal structure. n) Crystal structure of FeSn_2_. o,p) Crystal structures of Fe_0.74_Sn_5_ and FeSn_2_ from [001] direction. Reproduced with permission.^78^ Copyright 2011, American Chemical Society.

The inactive metals can alleviate the internal stress and promote the kinetics, however, they also cut down the overall capacity of the anodes. Therefore, it is important to find Sn‐based intermetallic compounds with high Sn content. Han's group^78^, ^84^, ^102^ discovered MSn_5_ (M = Fe, Co, and Fe_0.5_Co_0.5_) series intermetallic phases with stoichiometric structural vacancies. Fe_0.74_Sn_5_, Co_0.83_Sn_5_, and Fe_0.35_Co_0.35_Sn_5_ exhibit the highest theoretical capacities (>917 mA h g^−1^) among the Sn‐based binary and ternary alloys (M are inactive). The MSn_5_ (M = Fe, Co, and Fe_0.5_Co_0.5_) were synthesized by using Sn nanospheres as templates through a modified polyol process (**Figure** **7**a). As shown in Figure 7b–j, the as‐synthesized MSn_5_ compounds had uniform 30–50 nm spherical morphology with 3–5 nm amorphous surface oxide layers. The structural solution result revealed that the MSn_5_ (M = Fe, Co, and Fe_0.5_Co_0.5_) phases have the same crystal structure, belonging to the tetragonal system in the *P4/mcc* space group (Figure 7k,l). Fe_0.74_Sn_5_ was supposed to be the intermediate metastable phase between Sn and FeSn_2_ (Figure 7m–p). Likewise, Co_0.83_Sn_5_ could be considered as the intermediate phase between Sn and CoSn_3_.

**Figure 8:**
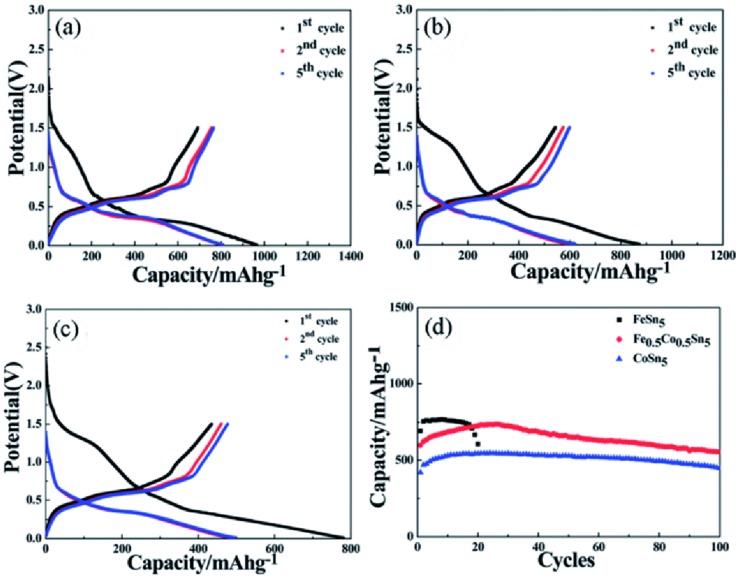
a–c) Voltage–capacity curves of FeSn_5_, Fe_0.5_Co_0.5_Sn_5_, and CoSn_5_, respectively. d) Dependences of charging capacities of MSn_5_ (M = Fe, Co, and Fe_0.5_Co_0.5_) on the cycle number at 0.05 C between 0.05–1.5 V. Reproduced with permission.^102^ Copyright 2015, Royal Society of Chemistry.

**Figure** **8** displays the electrochemical performances of MSn_5_ (M = Fe, Co, and Fe_0.5_Co_0.5_) intermetallic nanospheres. In despite of the same crystalline structure, MSn_5_ (M = Fe, Co, and Fe_0.5_Co_0.5_) had quite different electrochemical performances: FeSn_5_ exhibited a high capacity of about 750 mA h g^−1^, but decayed rapidly after 15 cycles; CoSn_5_ had a relatively low capacity of 500 mA h g^−1^, but cycled steadily up to 100 cycles; Fe_0.5_Co_0.5_Sn_5_ combined the advantages of high capacity of FeSn_5_ and good cyclability of CoSn_5_, exhibiting 736 mA h g^−1^ and still maintaining 92.7% after 100 cycles.

**Figure 9:**
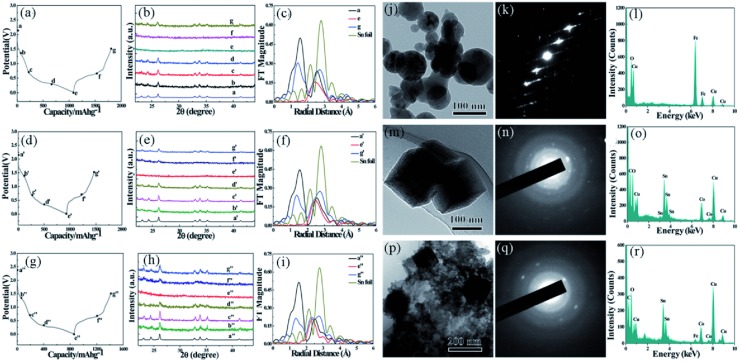
a,d,g) Voltage–capacity curves of FeSn_5_, Fe_0.5_Co_0.5_Sn_5_, and CoSn_5_ intermetallic compounds for the first cycle at 0.05 C. b,e,h) Synchrotron ex situ XRD patterns of FeSn_5_, Fe_0.5_Co_0.5_Sn_5_, and CoSn_5_ electrodes at different lithiation/delithiation potentials. c,f,i) Synchrotron XAFS profiles of MSn_5_ (M = Fe, Co, and Fe_0.5_Co_0.5_) electrodes at different lithiation/delithiation potentials. j,m,p) TEM, k,n,q) SAED patterns, and l,o,r) element EDS images of FeSn_5_, CoSn_5_, and Fe_0.5_Co_0.5_Sn_5_ intermetallic nanospheres after 100 cycles. Reproduced with permission.^102^ Copyright 2015, Royal Society of Chemistry.

In order to understand the lithiation/delithiation mechanism of MSn_5_ alloys, ex situ XRD and XAFS analyses were carried out. The result illustrated that these intermetallic compounds all showed reversibility during first lithiation/delithiation (**Figure** **9**a–i). However, as shown in Figure 9j,m,p, after 100 cycles the electrode materials had quite different morphological features. The FeSn_5_ anode turned to nanospheres of tens to hundreds nanometers; but the CoSn_5_ anode significantly changed to cubic structure; Fe_0.5_Co_0.5_Sn_5_ anode had both nanospheres and cubic structure. Remarkably, the EDS result and SAED patterns indicated that the Fe and Sn elements in FeSn_5_ were completely separated, and only Fe was detected in the investigated region (Figure 9k,l). In contrast, Sn and Co were still combined to form Co–Sn alloys in CoSn_5_ and Fe_0.5_Co_0.5_Sn_5_ (Figure 9n,o,q,r), which might explain the good cycling stability of CoSn_5_ and Fe_0.5_Co_0.5_Sn_5_. And the high electrochemical properties and kinetics of Fe was supposed to be accountable for the high capacity of FeSn_5_.

### 3.2 Sn Alloyed with Electrochemically Active Metals

In contrast to electrochemically inactive metals, electrochemically active metals can contribute to the overall capacity of the electrode. Furthermore, the alloyed elements always have discrepant discharge potentials with Sn. The temporally separated discharge processes promise that the Sn and active metals can alternately work as volume buffer agents. Benefiting from these synergistic effects, the Sn‐based alloys usually exhibit merits of both high specific capacity and long cycling life.^103^, ^104^ Up to now, many Sn‐based compounds alloyed with active elements have been investigated as potential anode materials, including Ge‐Sn,^105^, ^106^, ^107^ Sb–Sn,^108^, ^109^, ^110^, ^111^, ^112^ Ag–Sn,^113^, ^114^, ^115^ Mg–Sn,^116^, ^117^, ^118^ etc.

Xiao et al.^112^ synthesized SnSb/C nanocomposites by high‐energy mechanical milling (HEMM) and demonstrated them as high‐performance Na‐ion battery anodes. The sodiation/desodiation process of SnSb/C composites can be summarized as follows

(4) $$\mathrm{SnSb}\;\;+\;\;3{\mathrm{Na}}^{+}\;\;+\;\;3e^{-}\;\;↔\;\;{\mathrm{Na}}_{3}\mathrm{Sb}\;\;+\;\;\mathrm{Sn}$$

(5) $${\mathrm{Na}}_{3}\mathrm{Sb}\;\;+\;\;\mathrm{Sn}\;\;+\;\;3.75{\mathrm{Na}}^{+}\;\;+\;\;3.75e^{-}\;\;↔\;\;{\mathrm{Na}}_{3}\mathrm{Sb}\;\;+\;\;{\mathrm{Na}}_{3.75}\mathrm{Sn}$$

**Figure 10:**
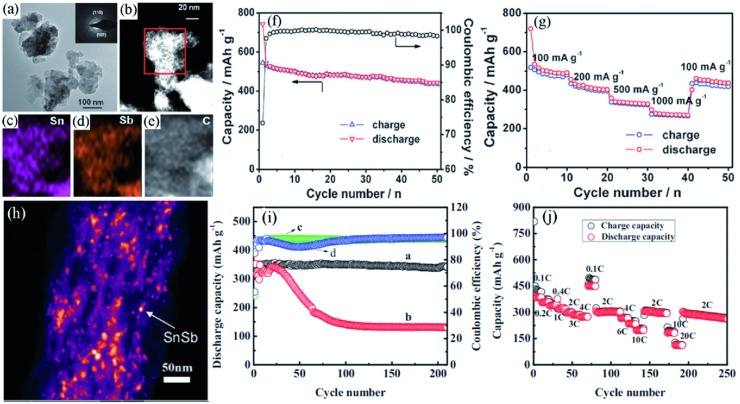
a) TEM image of SnSb/C nanocomposites, inset shows the corresponding SAED pattern. b–e) HAADF‐STEM image and corresponding EDS elemental mapping of SnSb/C. f) Cycling performance of SnSb/C electrode at 100 mA g^−1^. g) Rate capability of the SnSb/C electrode. Reproduced with permission.^112^ Copyright 2012, Royal Society of Chemistry. h) HRTEM image of porous CNF‐SnSb composites. i) Cycling performance and coulombic efficiency comparison of CNF‐SnSb electrodes in FEC‐containing and FEC‐free electrolytes at rate of 0.2 C. j) Rate capability of the CNF‐SnSb electrodes in FEC‐containing electrolyte. Reproduced with permission.^109^

As shown in **Figure** **10**a, the primary particle sizes of SnSb were about 10 nm, dispersing evenly in the carbon matrix. The tight combination of Sn, Sb, C elements ensured the excellent electrochemical performances (Figure 10b–e). In addition, the Sn‐ and Sb‐rich phases formed during sequential electrochemical reactions could self‐support one another, thus making the multicomponent alloy reaction materials ideal candidates for high‐performance battery anodes (Figure 10f,g).

By dispersing nano‐SnSb in porous carbon nanofiber (CNF), Ji et al.^109^ prepared CNF‐supported SnSb composites and evaluated their properties as anodes for SIBs (Figure 10h). They found that, similar to that in LIBs, FEC also played an important role in the stabilization of electrolyte in SIBs. FEC helped to suppress the reductive decomposition of the electrolyte and to form a thin, stable, and compact SEI film. This high‐quality SEI film improved the stability of the electrode and enhanced the kinetics of the Na^+^. As displayed in Figure 10i,j, the composites enabled a high reversible capacity of ≈350 mA h g^−1^, a long cycling life up to 200 cycles and excellent rate capability with 110 mA h g^−1^ retained at a high current rate of 10 000 mA g^−1^. In contrast, the CNF‐supported SnSb electrode in FEC‐free electrolyte exhibited serious capacity attenuation and low coulombic efficiency.

**Figure 11:**
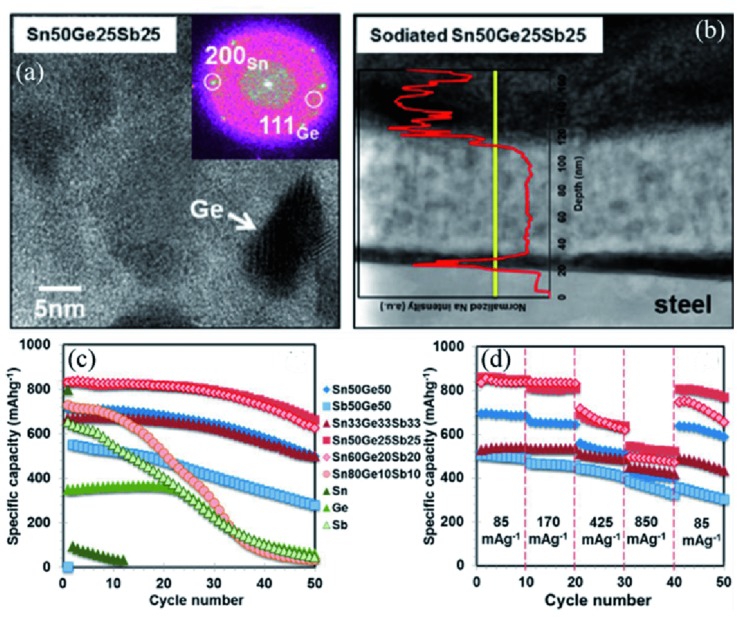
a) SAED and HRTEM images of Sn_50_Ge_25_Sb_25_ after 10 cycles. b) HAADF image and EELS line scan of Na distribution in Sn_50_Ge_25_Sb_25_ after the second sodiation. c) Cycling performance of the alloys and raw materials tested at 85 mA g^−1^. d) Rate performance of the alloys. Reproduced with permission.^120^ Copyright 2014, American Chemical Society.

Besides the binary alloys, ternary alloys are also wildly investigated as anodes in ion batteries.^119^, ^120^, ^121^, ^122^ Farbod et al.^120^ synthesized Sn–Ge–Sb series alloys by a co‐sputtering technique. Among all the alloys investigated, Sn_50_Ge_25_Sb_25_ showed the best cycling performance, which remained 662 mA h g^−1^ after 50 cycles (**Figure** **11**c). It also exhibited exquisite rate performance, delivering stable capacities of 658 and 381 mA h g^−1^ at 850 and 8500 mA g^−1^, respectively (Figure 11d). The existence of solutionized Sn could expand the lattice parameter of Ge beyond the equilibrium, which might allow facile nucleation of Na*_x_*Ge*_y_* phases with high Na content. As a result, the Ge in Sn–Ge–Sb could deliver a superior sodium storage ability compared to pure Ge (Figure 11a,b).

## 4 Structure Design of Sn‐Based Anode Materials

Reasonable structure design will introduce void space into the materials to accommodate the volume changes of Sn. The novel structural properties also improve both the structural stability and kinetics of the electrode materials. Therefore, structure design is a popular strategy to solve the issues facing Sn‐based anode materials. Herein, we have summarized the typical structure design advances of Sn‐based composites from 0D to 3D from recent years. As shown in **Table** **2**, we have classified these exquisitely designed Sn‐based composites with the focus on dimensional control, from 0D to 3D.

**Table 2:** Summary of the structure, electrochemical performance, and preparation method of some typical Sn‐based anode materials with reasonable structure design

Dimension	Sn‐based composite materials	Structure	Electrochemical performance	Preparation method
0D	Nano‐Sn/C composite^58^	≈10 nm Sn particles dispersed in the spherical carbon matrix	LIBs: 710 mA h g^−1^ after 130 cycles at 0.25 C, ≈600 mA h g^−1^ at 20 C	Aerosol spray pyrolysis
	Pitaya‐like Sn@C nanocomposites^56^	≈8 nm Sn particles dispersed in the spherical carbon matrix	LIBs: 910 mA h g^−1^ after 180 cycles at 200 mA g^−1^, 205.3 mA h g^−1^ at 16 000 mA g^−1^	Aerosol spray pyrolysis
	Sn@C nanocomposite^60^	≈5 nm Sn embedded in the carbon matrix	LIBs: 865.3 mA h g^−1^ after 200 cycles at 200 mA g^−1^	Hydrothermal method and postcalcination process
	Sn@C core–shell nanospheres^200^	Sn@C yolk–shell nanospheres with sprout‐like structure	LIBs: 430 mA h g^−1^ up to 500 cycles at 200 mA g^−1^	Chemical vapor deposition (CVD) method
	Sn@C nanoboxes^141^	Sn nanoparticles encapsulated in hollow carbon nanobox	LIBs: 810 mA h g^−1^ maintained after 500 cycles at 200 mA g^−1^	Sacrificial template method
	Sn@3D‐NPC^201^	Sn nanoparticles encapsulated in nanoporous carbon frameworks	LIBs: 740 mA h g^−1^ after 200 cycles at 200 mA g^−1^, 300 mA h g^−1^ at 5 A g^−1^	Thermal reduction
1D	Sn@carbon fibers^156^	Sn nanoparticles encapsulated in bamboo‐like hollow carbon fibers	LIBs: 737 mA h g^−1^ after 200 cycles at 0.5 C, 480 mA h g^−1^ at 5 C	Pyrolysis of nanofibers
	Sn QDs@CNFs^146^	Sn quantum dots embedded in N‐doped carbon nanofibers	LIBs: 887 mA h g^−1^ at 0.1 A g^−1^ after 200 cycles, 508 mA h g^−1^ at 0.4 A g^−1^ after 200 cycles	Electrospinning technology
	Sn NDs@PNC nanofibers^57^	Sn nanodots encapsulated in the porous carbon matrix	SIBs: 633 mA h g^−1^ at 200 mA g^−1^, 450 mA h g^−1^ at 10 000 mA g^−1^	Electrospinning and thermal treatment
	Sn–Al_2_O_3_–C^163^	Sn nanowires coated with Al_2_O_3_ and dispersed in carbon matrix	LIBs: 1063.3 mA h g^−1^ at 200 mA g^−1^ after 100 cycles	Mechanical pressure injection and ball milling
	TiO_2_‐Sn@CNFs^160^	Nano‐Sn dispersed in carbon nanofibers and TiO_2_ pipes	LIBs: 643 mA h g^−1^ at 200 mA g^−1^ after 1100 cycles SIBs: 413 mA h g^−1^ at 100 mA g^−1^ after 400 cycles	Electrospinning and atomic layer deposition
	Sn@C composite^153^	20–30 nm Sn particles dispersed in the porous carbon matrix	LIBs: 520 mA h g^−1^ after 100 cycles at 50 mA g^−1^, 155 mA h g^−1^ at 2 A g^−1^	Biotemplating method
	TiO_2_‐Sn/C arrays^167^	Sn encapsulated in TiO_2_ with carbon layer coated onto the surface	LIBs: 160 mA h g^−1^ after 100 cycles at 10 C, 90 mA h g^−1^ at 30 C	Hydrothermal method
2D	Sn@N‐RGO^183^	Sn nanoparticles encapsulated in N‐doped graphene sheets	LIBs: 481 mA h g^−1^ after 100 cycles at 0.1 A g^−1^, 307 mA h g^−1^ at 2 A g^−1^	Thermal reduction
	Sn@NG^180^	2–3 nm Sn nanoparticles embedded in the N‐doped graphene network	LIBs: 568 mA h g^−1^ at 1 A g^−1^ after 1000 cycles, ≈415 mA h g^−1^ at 3 A g^−1^	Carbonthermal reduction
	Sn–FSs^182^	Face‐to‐face sandwich structure clamped Sn sheets	LIBs: 650 mA h g^−1^ after 100 cycles at 100 mA g^−1^, 440 mA h g^−1^ at 2 A g^−1^	Carbonthermal reduction
	Sn/SnO/NGNSs^181^	Sn/SnO nanoparticles incorporated in crumpled N‐doped graphene nanosheets	LIBs: 853 mA h g^−1^ after 250 cycles at 1 A g^−1^, 241 mA h g^−1^ at 16 A g^−1^	Thermal treatment
3D	Sn@G‐PGNWs^196^	3D porous graphene networks anchored with 5–30 nm Sn nanoparticles	LIBs: 1089 mA h g^−1^ after 100 cycles at 0.2 A g^−1^, 270 mA h g^−1^ at 10 C	Chemical vapor deposition technique
	Sn‐MoS_2_‐C@C microspheres^202^	Sn nanoparticles embedded in MoS_2_ nanosheets with a thin carbon coating	SIBs: 580.3 mA h g^−1^ at 0.05 A g^−1^, 181.9 mA h g^−1^ at 5 A g^−1^, 245 mA h g^−1^ after 2750 cycles at 2 A g^−1^	Hydrothermal method
	Sn@C nanospheres on 3D layered carbon^192^	Sn nanoparticles encapsulated in carbon nanospheres and decorated on the 3D layered carbon	SIBs: cycled at 10, 20, 40, 80, and 10 mA g^−1^ for 20 cycles each	In situ carbonization and chemical vapor deposition techniques
	Graphene/Sn‐nanopillar^203^	Sn nanopillar arrays embedded between graphene sheets	LIBs: 679 mA h g^−1^ after 30 cycles at 0.05 A g^−1^, 408 mA h g^−1^ at 5 A g^−1^	Self‐assembly and film processing approaches
	Sn@CNTs‐VAGN^204^	Sn encapsulated in carbon nanotubes and displayed on vertically aligned graphene	LIBs: 1026 mA h g^−1^ at 0.25 C for more than 280 cycles, 140 mA h g^−1^ retained with a discharge time of 12 s	Microwave plasma irradiation reduction and in situ encapsulating techniques

### 4.1 0D Sn‐Based Structures

In contrast to bulk materials, 0D materials have ultra‐high specific surface area, which provides an abundance of reactive sites and improves the electrochemical activity of the electrode materials.^20^, ^129^ Benefiting from the small‐size effect, the volume expansion and particle aggregation of active materials are relieved.^58^, ^130^

**Figure 12:**
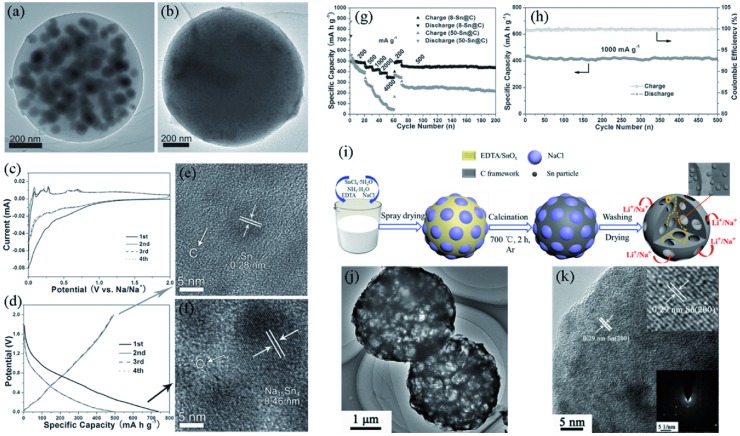
a,b) TEM images of 50‐Sn@C and 8‐Sn@C. c,d) CV and voltage–capacity curves of 8‐Sn@C. e,f) HRTEM images of 8‐Sn@C after first desodiation and sodiation process, respectively. g) Rate and cycling performance of the 8‐Sn@C and 50‐Sn@C electrodes between 0.01 and 2.0 V. h) Long‐term cycling performance of 8‐Sn@C at 1000 mA g^−1^. Reproduced with permission.^50^ i) Schematic illustration for the preparation process of Sn/NMCs. j,k) TEM, HRTEM images and SAED pattern of Sn/NMCs. Reproduced with permission.^134^ Copyright 2017, Royal Society of Chemistry.

The aerosol assisted method has been proven to be an effective strategy to produce 0D materials.^131^, ^132^ A series of 0D Sn/C composites have been successfully prepared by this method.^56^, ^58^, ^133^ As shown in **Figure** **12**a,b, nano‐Sn homogeneously embedded in carbon nanosphere composites were synthesized using aerosol spray pyrolysis method.^50^ This unique structure could restrain the volume fluctuation and suppress aggregation of Sn, thus effectively alleviating internal stress and prolonging the cycling life. When used as Na‐ion battery anodes, the 8 nm Sn samples (denoted as 8‐Sn@C) showed significantly better rate capabilities and cycling performances than that of 50‐Sn@C (Figure 12c–h).

In order to further enhance the kinetic properties of 0D Sn/C anode materials, our group^134^ designed Sn/N‐doped carbon microcage composites (Sn/NMCs) by using NaCl as a pore‐forming agent (Figure 12i). As the TEM image shows in Figure 12j, the composites had interconnected channels, which provided rapid ion‐transport paths. The ultra‐small Sn dots and flexible carbon matrix effectively improved the cycling stability (Figure 12k). Hence the Sn/NMCs exhibited both excellent cyclability and rate capability in Li^+^/Na^+^ batteries. The Sn/NMCs delivered 780 mA h g^−1^ at 200 mA g^−1^ and maintain 472 mA h g^−1^ after 500 cycles in LIBs, for SIBs, 439 mA h g^−1^ was achieved at 50 mA g^−1^ and 332 mA h g^−1^ was still maintained after 300 cycles.

**Figure 13:**
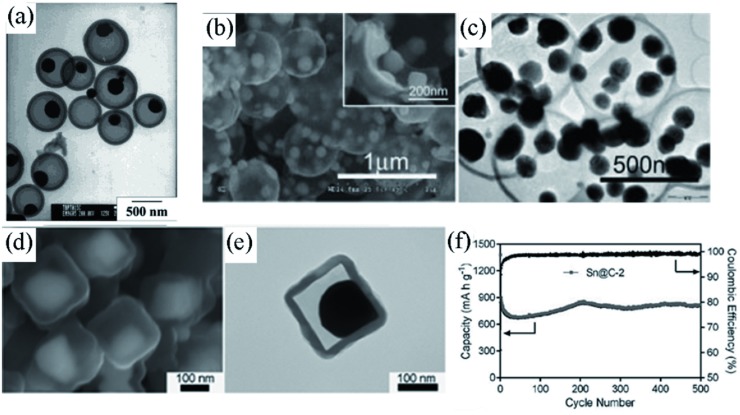
a) TEM image of Sn‐encapsulated spherical hollow carbon composite. Reproduced with permission.^139^ Copyright 2003, American Chemical Society. b,c) SEM and TEM images of TNHCs, respectively. Reproduced with permission.^140^ d,e) SEM and TEM images of Sn@C nanobox composite, respectively. f) Cycling performance of Sn@C nanobox composite at 200 mA g^−1^. Reproduced with permission.^141^

Yolk–shell architectures with inner buffering voids can provide reserved space to hold the expansion of active materials without destroying the protective sheaths, and the inner space is also beneficial to the rapid permeation of electrolyte.^135^, ^136^, ^137^, ^138^ Lee et al.^139^ synthesized Sn/C york–shell nanospheres with Sn particles encapsulated in hollow spherical carbon shells through a soft template method (**Figure** **13**a). The carbon shells acted as a barrier to prevent Sn particles from aggregation, and the hollow carbon capsule provided inner space to hold the volume change of Sn.

Similarly, Zhang et al.^140^ designed Sn nanoparticles encapsulated by hollow carbon spheres (TNHCs) through a hard template method. As shown in Figure 13b,c, multiple Sn particles less than 100 nm were sealed in carbon spherical shell with a thickness of about 20 nm. However, the capacity of TNHCs faded rapidly in the incipient 50 cycles, which might be ascribed to the pulverization and agglomeration of inner Sn particles.

Recently, Zhang et al.^141^ synthesized yolk–shell Sn@C nanobox composites (Figure 13d,e), they demonstrated that the thickness of carbon nanobox shell had a significant impact on the electrochemical performance. By optimizing the shell thickness, the Sn@C nanobox could maintain a reversible capacity of 810 mA h g^−1^ after 500 cycles, corresponding to 90% of the initial capacity (Figure 13f).

### 4.2 1D Sn‐Based Structures

Generally, 1D materials are capable of restraining the stress accumulation in the radial direction, and the ample internal space of 1D electrode materials can facilitate the stress relief. As a result, the 1D materials are promised to have good cycling stability. Moreover, the intertwined 1D network structures can promote the charge‐transfer process and improve rate performance. Up to now, many 1D Sn‐based materials have been fabricated to be applied as anodes, such as 1D nanowires,^19^, ^57^, ^59^, ^142^, ^143^, ^144^, ^145^, ^146^, ^147^, ^148^, ^149^, ^150^, ^151^, ^152^, ^153^, ^154^, ^155^ 1D nanotubes,^156^, ^157^, ^158^, ^159^, ^160^ and 1D nanoarrays,^5^, ^161^, ^162^, ^163^, ^164^, ^165^, ^166^, ^167^ etc.

**Figure 14:**
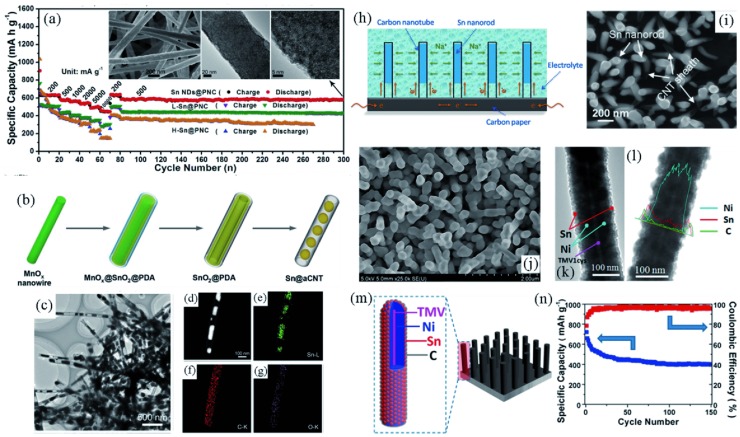
a) Rate and cycling performance of Sn NDs@PNC, L‐Sn@PNC (with lower Sn content), and H‐Sn@PNC (with higher Sn content); inset shows the SEM, TEM, and HRTEM images of Sn NDs@PNC after 300 cycles. Reproduced with permission.^57^ b) Schematic illustration of the preparation of Sn@aCNT composite. c–g) TEM, STEM, and corresponding elemental mapping images of Sn@aCNT composite. Reproduced with permission.^158^ h,i) Schematic illustration and SEM image of the free‐standing Sn@CNT‐CP electrode. Reproduced with permission.^165^ Copyright 2015, Elsevier. j–l) SEM, TEM, and EDS line scan mapping images of C/Sn/Ni/TMV1cys anodes. m) Schematic illustration of the C/Sn/Ni/TMV1cys anode. n) Cycling performance of C/Sn/Ni/TMV1cys anodes in Na‐ion batteries. Reproduced with permission.^161^ Copyright 2013, American Chemical Society.

The electrospinning technique possesses unique advantages to produce 1D nanofibers and is extensively used in fabricating 1D anode materials.^168^, ^169^, ^170^, ^171^ As shown in **Figure** **14**a, Sn nanodots (1–2 nm) finely encapsulated in porous N‐doped carbon nanofibers was prepared by Liu et al.^57^ using the electrospinning technique. These nanofibers could be used as current collector‐ and binder‐free anodes in SIBs. Attributed to the high Sn content (>60%), Sn NDs@PNC nanofibers exhibited a high reversible capacity of 633 mA h g^−1^ at 200 mA g^−1^, and still maintained 483 mA h g^−1^ at an extra high rate of 10 000 mA g^−1^. This exciting sodium storage performance was benefited by the remarkably uniform distribution of ultra‐small Sn nanodots in the carbon nanofiber matrix, as well as the kinetics enhancement by the N‐doped carbon frame.

With the advantages of ample internal cavity, high conductivity and high flexibility, carbon nanotubes (CNTs) are suitable containers for anode materials with high volume expansion rates. Zhou et al.^158^ reported a facile templating synthesis of Sn@aCNT composite with ≈70 nm Sn particles encapsulated in amorphous CNTs (Figure 14c–g). With the protection of the robust CNTs, the 1D Sn@CNT afforded excellent cycling stability and high rate capability. 749 mA h g^−1^ could be achieved at 200 mA g^−1^, and the high‐rate capability was also demonstrated when 573 mA h g^−1^ up to 500 cycles was attained at 1 A g^−1^. Besides carbon, other materials with high mechanical strength and stability can be also used as protective sheaths, such as TiO_2_ and Al_2_O_3_.^160^, ^163^, ^172^

1D nanoarray structures are advantageous in application for LIBs and SIBs. First, the integrated materials can be used as self‐supported electrodes without currents collector or binder. Second, the high length/radius ratio of the nanorods and the sufficient contact between active materials and the conductive substrates promises an efficient charge‐transfer process. Third, the interdigital spaces ensure the rapid and thorough infiltration of electrolyte and provide sufficient buffer zone to accommodate the volume fluctuation.

Xie et al.^165^ grew Sn@carbon nanotube (Sn@CNT) nanopillars vertically on carbon paper (denoted as Sn@CNT‐CP) using a facile soaking‐chemical vapor deposition technique (Figure 14h,i). Owing to the unique hierarchical architecture, the Sn@CNT‐CP delivered an initial reversible capacity of 887 µA h cm^−2^ when used as a free‐standing electrode in SIBs. When matched with an Na_0.80_Li_0.12_Ni_0.22_Mn_0.66_O_2_ cathode to assemble an Na‐ion full cell, it could sufficiently power an LED light.

Creatively, bioinorganic materials could be used as templates to synthesize nanoarray anodes. By depositing Sn onto an Ni‐coated tobacco mosaic virus (TMV) template and further introducing a thin layer of carbon over Sn, Liu et al.^161^ created novel C/Sn/Ni/TMV1cys anodes for SIBs (schematically illustrated in Figure 14m). Benefiting from the advanced hierarchical structure and the coated carbon layer (Figure 14 j,l), the aggregation and pulverization of Sn could be effectively alleviated; and the Ni sublayer was capable of establishing highly conductive passages. The C/Sn/Ni/TMV1cys nanoforest anodes delivered an initial reversible capacity of 722 mA h (g Sn)^−1^ and retained 405 mA h (g Sn)^−1^ after 150 deep cycles for SIBs (Figure 14n).

### 4.3 2D Sn‐Based Structures

Triggered by the discovery of graphene, 2D materials have attracted great attention from researchers.^173^, ^174^, ^175^, ^176^ 2D layered materials can effectively decrease the volume expansion and supply an abundance of reactive sites. In addition, the interlayer space of layered materials is beneficial to the full infiltration of electrolyte and inhibition of particles smash and aggregation.

**Figure 15:**
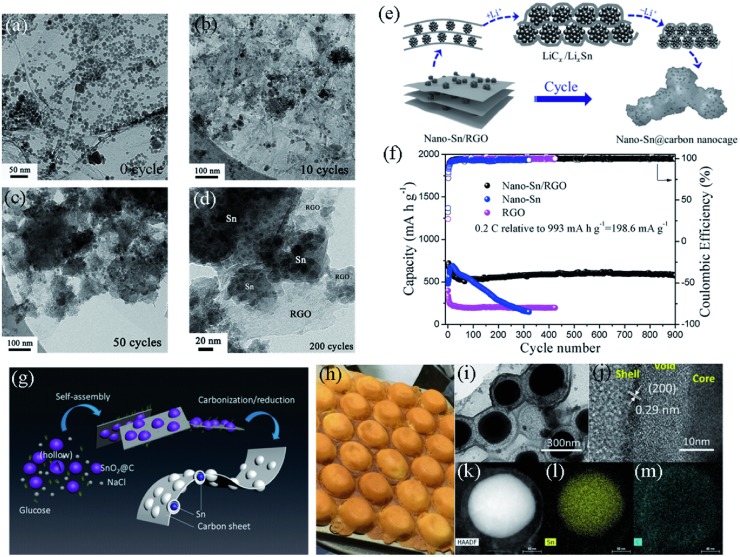
a–d) TEM images of nano‐Sn/RGO composites before and after 10, 50, and 200 cycles. e) Schematic illustration of the morphology evolution of nano‐Sn/RGO composite during cycling. f) Cycling performance comparison of nano‐Sn/RGO, nano‐Sn, and RGO. Reproduced with permission.^186^ g) Schematic illustration of the synthetic procedure of SCE. h) Picture of eggette. i,j) TEM and HRTEM images of SCE. k–m) STEM image and corresponding EDX elemental distribution of Sn and C. Reproduced with permission.^7^ Copyright 2016, American Chemical Society.

Owning to the remarkable features of high conductivity, high flexibility and structural strength, high surface area and ample functional groups, graphene has been demonstrated to be an excellent supporting matrix for Sn‐based materials.^174^, ^177^, ^178^, ^179^, ^180^, ^181^, ^182^, ^183^, ^184^, ^185^ Our group^186^ prepared nano‐Sn/reduced graphene oxide composite (nano‐Sn/RGO) with ≈10 nm Sn particles distributed on the surface and in the interface of layered RGO. As shown in **Figure** **15**a–d, we found that the nano‐Sn/RGO composite underwent a structural evolution during cycling (the morphological evolution is schematically illustrated in Figure 15e). The Sn@C spongy structure formed after about 50 cycles and was still retained even after 200 cycles, indicating the great stability of the Sn@C structure. Hence, the composite displayed a steady cycle up to 900 cycles at 0.2 C, in a sharp contrast to the poor cyclability of nano‐Sn (Figure 15f).

Furthermore, 2D/2D composite is also a feasible model for the modification of Sn anodes. Luo et al.^187^ synthesized graphene‐confined Sn nanosheets (G/Sn/G) through an elaborately designed glucose assisted method. The glucose‐derived carbon served not only as a protective layer to the liquid Sn but also as graphene‐like carbonaceous sheets for completing the encapsulation of Sn nanosheets. Attributed to the novel structure with surface‐to‐surface integration of graphene and Sn nanosheets, this composite exhibited enhanced performance. However, the cycling stability of G/Sn/G needed to be further improved.

Nevertheless, 2D materials usually have open architectures, which may be adverse to the immobilization of Sn particles. In order to combine the advantages of 2D structure and yolk–shell structure, Li et al.^7^ synthesized yolk–shell Sn@C eggette‐like compounds (SCE) through hydrothermal and self‐assembly processes (Figure 15g). As shown in Figure 15i–m, the SCE consisted of Sn cores encapsulated by carbon membrane networks, with extra voids between the Sn cores and carbon shells. The carbon capsules fixed the Sn particles and supplied buffer space for volume change. Furthermore, the carbon membrane networks could protect the Sn from aggregation as well as provide an abundance of reactive sites.

### 4.4 3D Sn‐Based Structures

3D materials usually combine the advantages of lower dimension materials, such as the ample inner space, high‐efficiency transport passages for ions and electrons, high specific surface area, and so on. Up to now, people have designed many 3D anode structures, such as 3D nanostructures,^188^, ^189^ 3D porous structures,^190^, ^191^, ^192^, ^193^ 3D network structures,^194^, ^195^, ^196^, ^197^, ^198^ etc.

**Figure 16:**
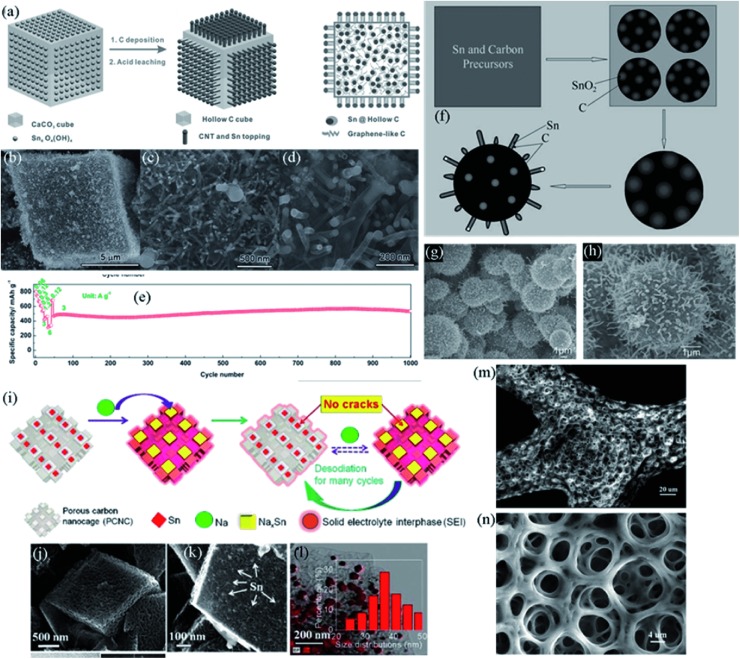
a) Schematic illustration of the synthetic procedure of the Sn/C composite. b–d) SEM images of Sn/C composite. e) Rate capability and cycling performance of the Sn/C composite. Reproduced with permission.^189^ f) Schematic illustration for the fabrication procedure of rambutan‐like Sn@C composite. g,h) SEM images of rambutan‐like Sn@C composite. Reproduced with permission.^188^ i) Schematic illustration of volume expansion cushioning by the PCNCs during cycling. j–l) SEM, high‐magnification SEM, and TEM elemental mapping images; the inset shows the size distribution statistics of Sn nanoparticles. Reproduced with permission.^190^ Copyright 2016, Elsevier. m) SEM image of an ESD‐derived film. n) SEM image of the as‐synthesized film after heat treatment at 900 °C. Reproduced with permission.^194^

3D nanostructure materials always have exquisite constructions, which are integrated by components with lower dimensions. For example, as shown in **Figure** **16**f–h, Deng and Lee^188^ reported a rambutan‐like Sn@C composite, composed of 0D Sn‐containing carbon mesospheres, 1D Sn@C nanotubes and quasi 1D Sn@C pear‐shaped nanoparticles. Similarly, Huang et al.^189^ designed a hierarchical tin/carbon composite using coprecipitation method to fabricate cubic particles, followed by chemical vapor deposition (CVD) of carbon and leaching with dilute HCl to remove the CaO which came from the decomposition of CaCO_3_ cube template (Figure 16a). As the SEM images show in Figure 16b–d, the as‐prepared Sn/C composite consisted of a microsized hollow cube, with CNTs rooted on the exterior surface of the hollow cube, while Sn nanoparticles were either encapsulated in carbon cubes or decorated on the tips of the CNTs. The rich voids inside the composite helped to alleviate mechanical stress, and the interlaced CNTs ensured rapid transfer of ions and electrons. As a result, this robust Sn/C composite showed a high capacity of 786.4 mA h g^−1^ at 60 mA g^−1^, and remarkably, delivered 537 mA h g^−1^ without obvious decay up to 1000 cycles at 3000 mA g^−1^ (Figure 16e).

Furthermore, to conquer the pulverization problem caused by huge volume changes, people also designed 3D porous Sn‐based materials.^6^, ^199^ The porous substrate accelerates ion transmission and supplies space for volume changes. The calculated sodiation voltage of Sn is around 0.15 V lower than lithiation potential, and the size of Na^+^ is much bigger than that of Li^+^, suggesting a larger buffer space is needed for porous materials in SIBs. Chen et al.^190^ developed a graphitic porous carbon nanocage‐Sn (PCNCs‐Sn) composite by a template‐assisted CVD method. Figure 16j–l show the SEM and TEM elemental mapping images of PCNCs‐Sn. When applied as SIB anodes, the PCNCs worked as shelters to prevent the Sn particles from smash and aggregation (schematiclly illustrated in Figure 16i). Theoretical calculations indicated that strong bonds between amorphous carbon and Na_15_Sn_4_ would form during cycling, which prompted the pulverization of Sn and deteriorated the conductivity, hence graphitic carbon was much more beneficial to the cyclability of Sn than amorphous carbon. The PCNCs‐Sn exhibited a high reversible capacity of 828 mA h g^−1^ at 40 mA g^−1^, good rate capabilities up to 2560 mA g^−1^, and long cycle life up to 1000 cycles.

3D skeleton structures are tenacious in resisting the impact of volume fluctuations, and the interconnected networks are beneficial to the kinetics. For instance, Li et al.^194^ fabricated a 3D porous core–shell Sn@C anode on a nickel foam substrate using electrostatic spray deposition (ESD) technique. This Sn@C composite had a fractal structure with small skeletons interconnecting to form a big framework (Figure 16m,n), this structure was robust and with the protection of the carbon layer, the 3D network anode showed enhanced electrochemical performance in lithium‐ion batteries.

## 5 Conclusions and Perspective

Lithium‐ion batteries have been booming for decades due to their advantages of high capacity density, long lifetime, rational working voltage, and environmental friendliness. They are gradually occupying the market of energy storage devices. Recently, sodium‐ion batteries have also drawn the extensive attention of research and development because of the abundance of sodium sources. The energy density of current commercial LIBs is still far from satisfactory. Besides the relatively low capacity of present commercially used cathodes, that of the present commercially used graphite anode (372 mA h g^−1^), is also too low to satisfy the universal application in EVs, wearable electronic devices, smart grid systems, etc. Sn‐based anode materials are supposed to be possible substitutes for graphite, and are also intensely relevant in the research of SIBs, with fairly high lithium and sodium specific capacities. In addition, small‐scale commercialization of the amorphous Sn–Co–C anode by Sony further ignited the study enthusiasm of Sn. However, the serious capacity attenuation resulting from the drastic volume fluctuations still predominantly hinders the practical utilization of Sn anodes. To overcome the challenges of particle pulverization and low coulombic efficiency, researchers have devoted much effort to the modification and architectural design of Sn‐based anodes.

The typical optimization strategies of Sn anode include nanocrystallization, modification with a carbon matrix, alloying with other metals, structure design, and so on. Reducing Sn particle sizes to nanoscale is effective in prolonging the cycling life of pure Sn anodes. On one hand, the nanoscale effect can essentially reduce the absolute volume expansion of a single particle and mitigate the inner strain, hence retarding particle pulverization. On the other hand, the plentiful clearance space among nanosized materials offers relief area for volume change, as well as accelerates infiltration of electrolyte and diffusion of ions, thereby, insuring the structural integrity of the electrode and significantly improving the kinetic properties. However, the aggregation nature of Sn weakens the efficacy of nanocrystallization. In order to inhibit the aggregation of Sn, people try dispersing nano‐Sn in flexile matrixes, especially carbon. The carbon matrix can restrain the Sn particles from coalescence and growth during both material preparation and the electrochemical cycling process. It was found that the choice of alloying metals is vital to the electrochemical performance of Sn‐based alloys. The inactive metals construct efficient conductive networks and volume buffer zones for Sn, while the active metals not only contribute to the overall capacity of anode materials, but also serve as self‐support buffer agents alongside Sn. The electrochemical performances of Sn‐based alloys are synthetically dependent on the crystal structure of the alloys, binding force and the kinetic characteristics of alloying metals. Furthermore, the exquisite structure design is quite useful in obtaining high‐performance Sn‐based anode materials and is favored by researchers. Up to now, a series of novel Sn composite structures have been exploited, ranging from 0D to 3D. The advanced structures always introduce rational void space into the materials to accommodate the volume changes, and/or build electric conduction networks and ion transport channels. Therefore, the Sn‐based composites with elaborately designed structures always show enhanced electrochemical performances in ion batteries.

In future studies, the volume variation, aggregation, and superfluous SEI formation are still the main problems that need to be solved. From the industrialized application point of view, we believe that metallic Sn‐based/carbon composites (including Sn/C, Sn‐based alloys/C) have the greatest prospects. The carbon matrix can effectively prevent the shedding and aggregation of active materials, relieve the volume expansion of active materials and improve the electrical contact. Furthermore, the carbon matrix protects the active materials from direct exposure to electrolyte and suppresses the excessive formation of SEI film. The preparation methods of metallic Sn‐based/carbon composites are wide and suitable for industrialization. From the scientific research point of view, special and exquisite architectural designs will continue to be favored by researchers. These well‐designed metallic Sn‐based materials are effective in improving the electrochemical performance and studying the optimization mechanisms. However, the complex and costly preparation methods limit the practical application. Besides nanomaterials, micromaterials may become a new tendency for the study of IVA group anodes. The micromaterials can improve the coulombic efficiency and tap density effectively, but the poor cycling stability is still the main drawback.

After years of research, the electrochemical performances of metallic Sn‐based alloy anodes have been dramatically improved through a series of modification strategies. However, some key issues such as pulverization, and lithium/sodium sources loss are still unsolved. Finally, development of large‐scale industrial preparation of Sn‐based anode materials is still required for the commercial application of Sn‐based materials. Despite there are many challenges, the Sn‐based materials are still believed to hold great potential in developing next‐generation high‐performance ion battery anodes. We expect that this review article can supply some reference and inspiration for the development of Sn‐based anode materials.

## Conflict of Interest

The authors declare no conflict of interest.
